# Revealing bovine schistosomiasis in Malawi: Connecting human and hybrid schistosomes within cattle

**DOI:** 10.1016/j.onehlt.2024.100761

**Published:** 2024-06-14

**Authors:** Alexandra Juhász, Peter Makaula, Lucas J. Cunningham, Sam Jones, John Archer, David Lally, Gladys Namacha, Donales Kapira, Priscilla Chammudzi, E. James LaCourse, Edmund Seto, Sekeleghe A. Kayuni, Janelisa Musaya, J. Russell Stothard

**Affiliations:** a Liverpool School of Tropical Medicine, Liverpool, UK; b Semmelweis University, Budapest, Hungary; c Malawi Liverpool Wellcome Research Programme, Blantyre, Malawi; d Kamuzu University of Health Sciences, Blantyre, Malawi; e University of Washington, Seattle, WA, USA

**Keywords:** Urogenital schistosomiasis, *Schistosoma haematobium*, *Schistosoma mattheei*, Hybridisation, One Health

## Abstract

In Malawi, the putative origin of a newly described *Schistosoma haematobium*-*mattheei* hybrid human schistosome was assessed upon a seminal molecular parasitological survey of cattle. Using miracidia hatch test (MHT) and carcass inspection at slaughter, mean prevalence of bovine schistosomiasis was 49.1% (95% CI: 43.7–54.6%) and 10.3% (95% CI: 6.0–16.2%) respectively, though significant spatial heterogeneity was noted. Approximately 2.0% of infected cattle, and only those from Mangochi District, shed *S. haematobium*-*mattheei* and/or *S. haematobium* in faeces. To quantify schistosome (re)infection dynamics, where a *S. haematobium*-*mattheei* hybrid was present, we undertook a novel pilot GPS-datalogging sub-study within a specific herd of cattle (*n* = 8) on the Lake Malawi shoreline, alongside a praziquantel (40 mg/kg) treatment efficacy spot check. At sub-study baseline, all GPS-tagged cattle had proven daily water contact with the lake. Each animal was patently infected upon MHT, with older animals shedding less miracidia. At one month review, whilst parasitological cure was 100.0%, from six weeks onwards, (re)infection was first noted in the youngest animal. By three-month review, all animals were patently (re)infected though only miracidia of *S. mattheei* were recovered, albeit in much lower numbers. To conclude, infection with *S. mattheei* is particularly common in cattle and demonstrates a previously cryptic burden of bovine schistosomiasis. Within Mangochi District, bovine transmission of both *S. haematobium*-*mattheei* hybrids and *S. haematobium* are now incriminated, with unequivocal evidence of contemporary zoonotic spill-over. Future control of urogenital schistosomiasis here in the southern region needs to develop, then successfully integrate, a One Health approach with appropriate mitigating strategies to reduce and/or contain bovine schistosomiasis transmission.

## 1 Introduction

Across sub-Saharan Africa (SSA), control of urogenital schistosomiasis, caused by the human blood fluke *Schistosoma haematobium*, features prominently within the World Health Organization's (WHO) 2021–2030 Roadmap for Neglected Tropical Diseases (NTDs). Its prominence was boosted, in part, by recent calls to alleviate female genital schistosomiasis (FGS), a gender-specific manifestation where adult worms deposit tissue damaging eggs inside a woman's genital organs [1]. Another newly appreciated biological dimension of *S. haematobium* is its innate capacity, given sufficient opportunity, to hybridise with other schistosome species within the *S. haematobium* group [[2], [3], [4], [5], [6], [7], [8]]. Molecular characterisation of natural populations with genetic markers has revealed in West Africa, for example, various hybrid schistosomes with their transmission firmly tied to zoonotic inputs [9,10]. This changing appreciation in the emergence of *S. haematobium*-hybrids, particularly from livestock, has led to WHO's recent compendium on One Health approaches to broaden future dimensions in disease surveillance and control across Africa [11].

Even though viable hybrids between *S. haematobium* and *S. mattheei* have been known since 1980 from, experimental crosses in the laboratory [12], only in 2019 was this hybrid combination first confirmed in infected children in Malawi [10]. The molecular investigation to do so was initiated upon observations of morphologically atypical *S. haematobium* eggs in school-aged children's urine, to later reveal *S. haematobium-mattheei* and *S. haematobium-bovis* combinations in two districts, Mangochi and Nsanje, located in Southern Malawi [10]. Whilst a peripatetic distribution of *S. haematobium-bovis* could have been expected given its increasing reports elsewhere [13,14], the occurrence of *S. haematobium-mattheei* was unexpected. Whilst *S. mattheei* is a common parasite of wild and farmed ungulates in South Africa, and is known to infect people, it has never been reported in Malawi before [15]. The latter might simply arise from a dearth of surveillance for bovine schistosomiasis, despite having an active national control programme primarily engaged in administration of preventive chemotherapy to at-risk populations.

To shed new light on the putative emergence of *S. haematobium*-hybrids in people, we undertook a seminal molecular parasitological survey of Malawian cattle for bovine schistosomiasis, inclusive of a pilot sub-study that closely monitored, over a three month period, a small herd of cattle with GPS-dataloggers, before and after praziquantel treatment.

## 2 Materials and methods

### 2.1 Study area and study population

**Fig. 1:**
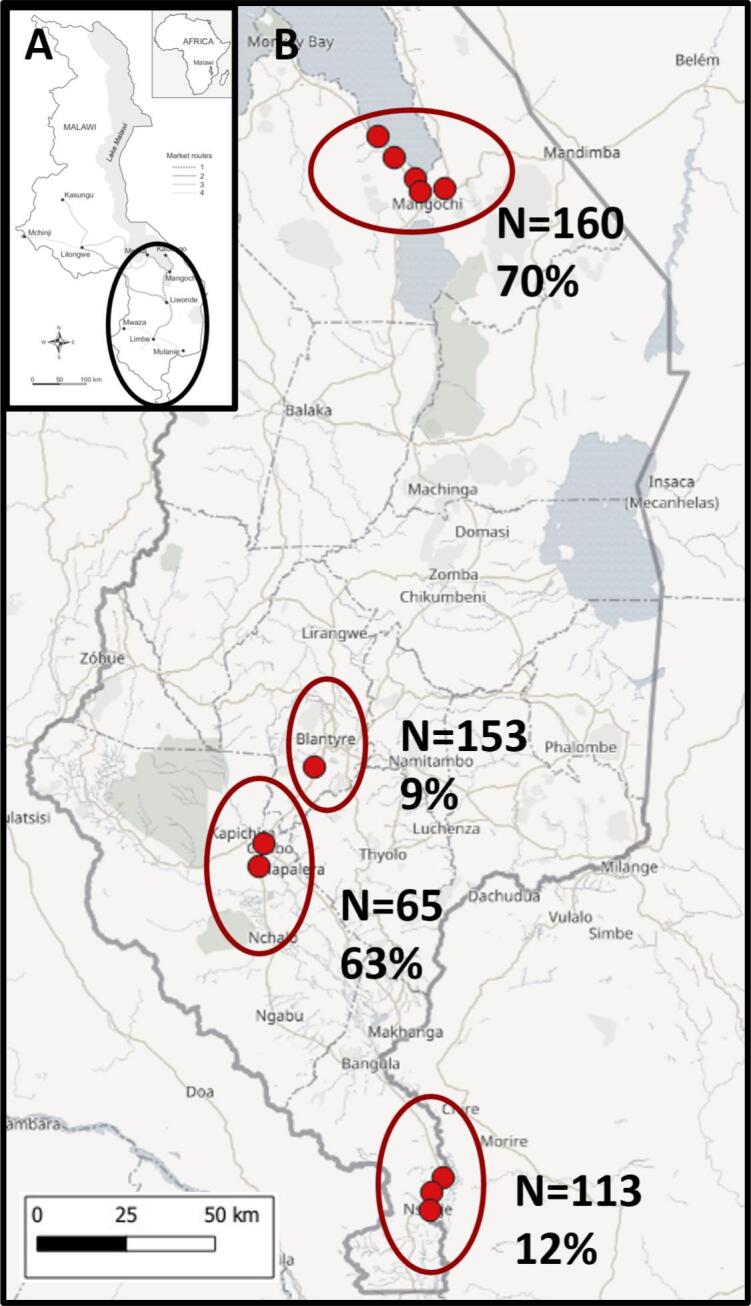
Sampling area. **A)** Map of Malawi; **B)** Map showing study sites and the prevalence of *Schistosoma* spp. infections in cows sampled from Malawi, South-eastern Africa from April 2022 to May 2022.

Our study was carried out at 11 inspection sites across three districts (Mangochi, Nsanje and Chikwawa) of Malawi (Fig. 1), as part of a larger project entitled “Hybridisation in urogenital schistosomiasis (HUGS)”. The HUGS study is concurrently screening local communities and intermediate host snails for hybrid schistosomes. We report here specifically on the prevalence and infection intensity of *Schistosoma* spp. in local cattle (*Bos taurus domestica*), as combined with genotyping adult worms and miracidia.

### 2.2 Faecal sample collection and diagnostics

Taken immediately after defecation, faecal samples from 338 individual cattle from nine locations, averaging 40 samples per inspected herd, were obtained. To estimate accurately the prevalence and intensity of infection, a combination of two diagnostic methods was used: i) the miracidial hatching test (MHT) [16] and ii) the sedimentation method (SD). All faecal samples were subjected to examination for the presence of live miracidia using the MHT, as previously described [17] using ∼15 g of bovine faecal sample. Individual miracidia were harvested using a P20 Gilson micropipette in 2.5 μL of water, being checked within the pipette tip, before placing on Whatman FTA® cards (GE Healthcare Life Sciences; Amersham, UK). If the MHT gave a positive result, the remaining faecal material was examined by sedimentation to visualise schistosome egg morphology by x100 microscopy to enable preliminary species identification.

### 2.3 Inspection of carcass at abattoir

In total, 155 carcasses of cattle were examined, inspections were undertaken at a commercial slaughterhouse in Blantyre and two other locations where spot slaughtering was performed. Collectively, this permitted an adequate test of prevalence of bovine schistosomiasis in slaughtered livestock. Adult worm pairs were separated in saline, inspecting intrauterine ova by x100 microscopy for preliminary species identification, with paired worms stored separately in 90% ethanol for later genotyping. For infected animals, a 15 g small intestine biopsy was taken: 1 g was processed with potassium hydroxide (KOH) digestion to visualise schistosome ova.

### 2.4 Molecular identification of miracidia and adult worms

#### 2.4.1 Miracidial DNA extraction from FTA cards

Miracidia stored on Whatman FTA cards were alkaline eluted to extract the genomic DNA for genotyping [10]. Briefly a 2 mm disc was each punched from the FTA card where miracidia were deposited, a clean section of the FTA card was similarly punched out to act as a negative extraction control, for every batch of FTA extractions. To each of the 2 mm discs 14 μL of solution 1 (0.1 M NaOH, 0.3 mM EDTA, pH 13.0) was added and the sample incubated at room-temperature for five minutes. After incubating, 26 μL of solution 2 (0.1 M Tris-HCL, pH 7.0) was added and the sample incubated for a further 10 min at room-temperature followed by pulse vortexing three times. After the final incubation step 2 μL of FTA sample was then used for downstream molecular analysis.

#### 2.4.2 Whole adult worm DNA extraction

Prior to undergoing DNA extraction adult worms were first cut in half, so as to not use all the tissue material and the half selected for DNA extraction then underwent washing with distilled water to remove any remaining ethanol. DNA from adult worms was then extracted using the QIAamp DNA Mini Kit (cat number: 51306, Qiagen) following the manufacturer's instructions with the addition of phocine herpes virus (PhHV) to act as an internal extraction and later amplification control.

#### 2.4.3 Hybrid identification with high resolution melt (HRM) profiling

A novel real-time PCR assay was developed for the rapid screening of both miracidia stored on FTA cards and the whole worm extracts, in order to identify samples of interest that could then be confirmed as hybrids using Sanger DNA sequencing. The real-time PCR assay consists of two separate reactions with the first reaction targeting the mitochondrial genome (mtDNA) and the second reaction targeting the nuclear genome (nDNA). Both the mtDNA and nDNA assays are capable of distinguishing between different schistosome species using high resolution melt (HRM) peak analysis. In brief, the mtDNA real-time PCR is a species-specific multiplex assay targeting the tRNA-Lys gene markers for *S. curassoni, S. bovis, S. haematobium* and *S. mansoni* and the ND6 and ND4 genes for *S. mattheei* and *S. margrebowiei* respectively.

All real-time PCR assays were carried out using a Magnetic Induction Cycler (MIC) PCR thermocycler (Bio Molecular Systems (BMS), UK). All real-time PCR assay plates were prepared using a MYRA liquid handling system (Bio Molecular Systems (BMS), UK). Reactions were carried out in 12 μL volumes and consisted of 6 μL of Type-it HRM supermix (Qiagen, Manchester, UK) 200 nM final concentration of primer and 2 μL of template, with any remaining volume being made up of nuclease free water. Cycling conditions consisted of an initial hold of 95 °C for five minutes followed by 35 three-step cycles of 95 °C for 10s, 58 °C for 30s and 72 °C for 10s. Following the 35 cycles, a final melt step was carried out, with temperatures ranging from 68 °C to 90 °C with a ramp rate of 0.1 °C/s. The novel HRM primers with corresponding melt temperatures (Tm) are presented in Table 1 and the HRM peak profiles are presented in supplementary material (Fig. 1).

**Table 1:** Sequences and average Tm values for the novel HRM mtDNA and nDNA real-time PCR assays.

mtDNA primers			
Target sp.	Primer name	Primer sequence	Product Tm (°C)
*S. mattheei*	SchMattF	GTTGGTTTCGTATTTTTTTATGTTAAGG	71.53
	SchMattR	CTAACTTAGCGCTTCACAAAATGC	
*S. curassoni*	SchCurrF	GTCGTGCTTTTGGTGATTAGC	72.47
	SchCurrR	CCTACGCCCGATAAACTAAAC	
*S. bovis*	SchBovF	CAACATAAGATGATTGTAGTTAGC	73.39
	SchBovR	CTTTATTACTCGGCCACGATATG	
*S. haematobium*	SchHmF2	GCTGTAAAGGTGGCTGATAGTAGC	75.55
	SchHmR2	TATCAACTTAACTATGCACCTAGTG	
*S. mansoni*	SchMnF	GGTTGAAGAGGAGGTTCGTG	76
	SchMnR	GGTCGCAATATACTCGACACC	
*S. margrebowiei*	SchMrgF2	GGATCACGAAGTTGGGCTATAC	77.8
	SchMrgR2	GAATATCAGCACAGACAATACTTGAAC	


nDNA primers			
Target sp.	Primer name	Primer sequence	Product Tm (°C)
*Schistosoma* sp.	NUC ITS F	GCTCGAGTCGTGGCTTAATGAC	80.02–82.18⁎
	NUC ITS R	CTGATCCGAGGTCRGGGTCAATTA	

⁎ The nDNA primers (NUC ITS F + R) produced the following Tm for each species: 80.02 °C (*S. mansoni*), 80.96 °C (*S. margrebowiei*), 81.06 °C (*S. mattheei*), 81.62 °C (*S. bovis*), 81.7 °C (*S. curassoni*) and 82.18 °C (*S. haematobium*).

#### 2.4.4 Sanger DNA sequencing of ITS and *cox*1 genes

Sanger DNA sequencing was used on a sub-set of samples identified using the real-time PCR as being samples of interest. These methods have been previously described by Huyse et al. (2009), however, in brief primers targeting the nuclear ribosomal ITS (ITSF: TAACAAGGTTTCCGTAGGTGAA & ITSR: TGCTTAAGTTCAGCGGGT) and *cox*1 (Asmit1F: TTTTTTGGTCATCCTGAGGTGTAT & SCHR: TAATGCATMGGAAAAAAACA) genes of *Schistosoma* spp.. These primer pairs amplified a 981 bp and 583 bp region of their respective target gene [2]. Reactions were carried out in a 25 μL reaction, comprising 12.5 Q5® High-Fidelity mastermix (New England BioLabs, Hitchin, UK), 400 nM of primer and 4 μL of template. PCR cycling conditions for the two assays consisted of an initial hold at 98 °C followed by 40 three-step cycles consisting of 98 °C for 10s, and annealing temperature of either 40 °C (ITS) or 54 °C (*cox*1) for 10s and an extension step at 72 °C for 30s. upon completion of the 40 amplification cycles a final extension step was carried out at 72 °C for 2 min. PCR products were then separated alongside 100 bp hyperladder (Scientific Laboratory Supplies, Nottingham, UK) in a 1% agarose gel stained using SYBR™ Safe DNA gel stain (Thermo Fisher Scientific, Paisley, UK).

#### 2.4.5 Pilot GPS datalogging and praziquantel treatment sub-study

As a pilot sub-study, the movements and relative water contact levels of eight cattle, a sub-herd within Mangochi 3, were recorded over a three-month period by GPS dataloggers (from May 2022 to July 2022). The technology behind the GPS fitted collars was adopted from dementia care technologies (TechSilver, 2022), providing regular spatial recording GPS updates on animals' movements, alerting a mobile phone device reporting every 5 min via the SPOT satellite system. Each GPS collar was marked with white paint with letters (A-H).

To increase diagnostic sensitivity faecal material (15 g) from the animals in the GPS sub-study was regularly inspected with the above described MHT (baseline, 1-, 4-, 6-, 8-, 12-weeks), upon three separate faecal samples retrieved per rectum. The pedigree relationships of the animals and the ages of the eight collared cattle were reported by the owners (Fig. 3 B). An estimate of weight for each animal was collected by chest measurement, also referred to as heart girth [[18], [19], [20]]. On 17th April 2022, all animals were treated with a single per oral dose of 40 mg/kg body weight of praziquantel (PZQ) (600 mg IDA Pharmacy).

## 3 Results

### 3.1 Analysis of cattle faeces

A total of 338 faecal samples were processed from the nine study sites. Miracidia were successfully hatched from 166 samples using the MHT; 160 cattle faeces were inspected upon the southern shoreline of Lake Malawi from four locations finding 70.6% were heavily infected (with an infection intensity of more than five miracidia per gram of faeces). By contrast, of the faecal samples from two sites in Chikwawa, 63.1% were infected, whilst there was 12.4% at three sites in Nsanje; with mean infection intensities of less than 5 miracidia per gram (Table 2, Fig. 1). A visual inspection analysis of schistosome egg morphology concluded that there were no unusually shaped eggs or eggs that could be considered as either *S. haematobium* or *S. mansoni*.

**Table 2:** Prevalence (%) of *Schistosoma* spp. in faecal samples of cows across our nine study sites.

	Coordinates		Cattle		Miracidia	*Schistosoma* spp.
Study site	South	East	N _MHT_⁎	No. positive	Mean No.	Prevalence (%) 95% CI
Mangochi 3	14.36915°	35.17623°	40	32	5.6	80.0 (65.2–89.5)
Mangochi 4	14.42255°	35.23224°	40	21	3.5	52.5 (37.5–67.1)
Mangochi 5	14.45145°	35.24238°	40	29	5.3	72.5 (57.2–83.9)
Mangochi 7	14.44386°	35.30623°	40	31	3.5	77.7 (62.5–87.7)
**Total from Mangochi**			**160**	**113**		**70.6 (63.2–77.1)**
Nsanje 3	16.85401°	35.29956°	40	1	4	2.5 (0.4–12.9)
Nsanje 4	16.88922°	35.27124°	40	4	1	10.0 (4.0–23.1)
Nsanje 5	16.92984°	35.26588°	33	9	6.1	27.3 (13.3–45.5)
**Total from Nsanje**			**113**	**14**		**12.4 (6.9–19.9)**
Chikwawa 1	16.04210°	34.84577°	40	24	1.3	60.0 (44.6–73.7)
Chikwawa 2	16.09739°	34.83376°	25	17	4.2	68.0 (48.4–82.8)
**Total from Chikwawa**			**65**	**41**		**63.1 (50.2–74.7)**
**Total examined**			**338**	**166**		**49.1 (43.7–54.6)**

⁎ Number of cattle sampled by miracidial hatching technique.

### 3.2 Inspection of cattle carcasses

A total of 155 slaughtered cattle from abattoirs were examined. The mean prevalence of *Schistosoma* adult flukes in cattle at Blantyre abattoirs was 9.8%. The presence of *Schistosoma* spp. was also noted in two slaughtered animals in Mangochi District (Table 2, Fig. 1).

### 3.3 Putative detection of hybrids with HRM real-time PCR

#### 3.3.1 Cattle miracidia FTA real-time PCR

A total of 613 miracidia FTA samples from 166 parasitologically positive cattle were processed using the duplex real-time PCR assays, targeting mtDNA and nDNA markers, averaging 3.7 samples per cow. Of the 613 samples screened 481 returned a positive result for mtDNA markers (Ct-cut off 36) and 480 produced positive results for the nDNA markers (Ct-cut off 36). Samples that produced a positive result for both mtDNA and nDNA markers numbered 455. The identification of a hybrid miracidia requires the comparison of both mtDNA and nDNA markers, therefore analysis was performed only on those 455 samples that produced positive mtDNA and nDNA results.

**Fig. 2:**
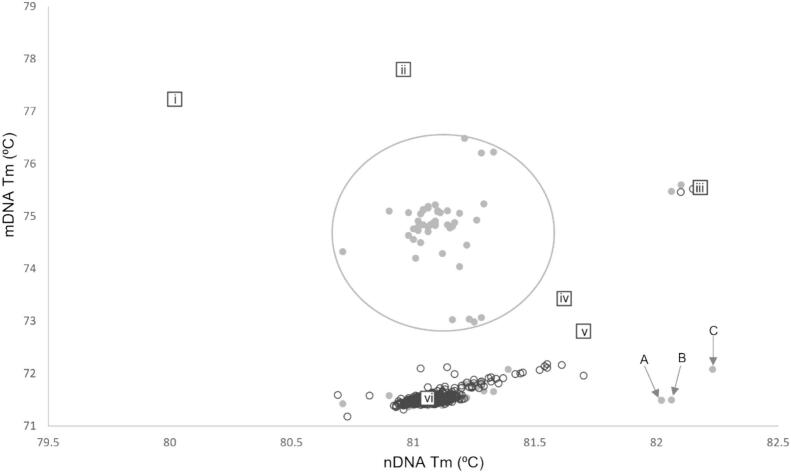
Bivariate graph plotting the mtDNA and nDNA melt-peak (Tm) results for miracidial FTA spots screened with the two-tube HRM assay. Square markers denote positive controls, derived from adult worms, for i) *S. mansoni*, ii) *S. margrebowiei*, iii) *S. haematobium*, iv) *S. bovis*, v) *S. curassoni* and vi) *S. mattheei*. Hollow circles represent positive samples that produced single peaks, solid grey circles represent double peaks. Circled cluster in the centre of the graph are those samples that produced a secondary melt peak that did not correspond to any of the melt-peaks of the control samples. The position of three out of the four putative-hybrid samples are shown with labels A (GPS-H), B (Chimbende 31a) and C (29AA9).

Of the 455 mtDNA/nDNA positive real-time PCR samples 405 (89.0%) produced a single melt-peak in both assays of which 402 (88.4%) clustered with the expected melt temperature (Tm) of *S. mattheei* (Table 1), three samples (0.4%) clustered with the expected Tm of *S. haematobium*. The remaining 51 (11%) samples produced double peaks either in the mtDNA assay (*n* = 48) or the nDNA assay (*n* = 2) or in both (*n* = 1). Of the samples with double-peaks four were putatively identified as *S. haematobium-mattheei* hybrids. Sample 31b from Mangochi site 7 produced double melt-peaks in both the mtDNA and nDNA real-time PCR assays clustering with *S. mattheei* and *S. haematobium* (Table 3). A single-peak in the mtDNA assay but a double peak, in the nDNA assay was produced by a sample from cow GPS-H, Mangochi site 4 and from sample 29AA9 from Mangochi site 3. The final sample, sample 31a, Mangochi site 7, produced a double mtDNA peak but a single ITS peak. The other 47 double-peak samples produced *S. mattheei* nDNA peaks and a secondary mtDNA peak that failed to cluster with expected species clusters (Fig. 2) (See Table 4.).

**Table 3:** Prevalence (%) of *Schistosoma* spp. in carcass of cows from Blantyre and Mangochi.

	Coordinates		Cattle			
Study site	South	East	N	No. positive	Estimated mean worm burden in the population (min-max)	*Schistosoma* spp. Prevalence (%) 95% CI
Blantyre	15.857540°	34.974230°	153	15	1–556	9 (5.6–15.75)
Mangochi	14.45145°	35.24238°	2	2	20–98	100 (20.7–100)
**Total examined**			**155**	**16**		10.3 (6.0–16.2)

**Table 4:** Results of basic local alignment of ITS and *cox*1 against NCBI GenBank database.

Sample ID	BLAST ID		Query cover (%)		Per identity (%)		*S. mattheei x S. haematobium* heterozygous peaks	
	ITS	*Cox*1	ITS	*Cox*1	ITS	*Cox*1	ITS	C*ox*1
Chimbende 31a	*S. haematobium*	*S. haematobium*	100	100	99.9	98.6	No	Yes
Chimbende 31b	*S. haematobium*	*S. mattheei*	100	100	99.9	99.5	No	Yes
Chimbende 33a	*S. haematobium*	*S. haematobium*	100	100	99.9	99.5	No	No
Chimbende 33b	*S. haematobium*	*S. haematobium*	100	100	99.9	99.5	No	No
GPS-H	*S. haematobium*	*S. mattheei*	100	100	99.9	99.8	No	Yes

Full alignments of a 900 bp ITS and 496 bp *cox*1 chromatograms for the forward primers can be found in.

Supplementary materials showing the heterozygous/mixed peaks of the hybrid samples.

The two pure mtDNA/nDNA *S. haematobium* samples originated from two cows (cow 31 and 33) at Mangochi site 7. The putative hybrid samples originated from Mangochi sites 3, 4, and 7. The double mtDNA and nDNA sample originated from Mangochi site 7 (cow 31). The remaining three putative samples originated from Mangochi site 7, cow 31, Mangochi site 4 GPS- H and cow 29AA9. No putative hybrids were detected in cows sampled from Nsanje and Chikwawa sites.

#### 3.3.2 Whole worm real-time PCR

Following DNA extraction 53 whole worms were screened using the mtDNA and nDNA real-time PCR assays, all worms produced single peaks in each respective assay. These melt-peaks clustered with the *S. mattheei* mtDNA and nDNA melt-peaks, indicating that all worms screened were pure *S. mattheei* males and females.

#### 3.3.3 Sanger DNA sequencing

Based on the real-time PCR mtDNA and nDNA assays six samples were sent for ITS and Sanger *cox*1 sequencing, these were the two pure *S. haematobium* samples (Chimbende cow 31a, 33b and 33b) alongside the four putative hybrid samples (Chimbende cow 31b, GPS-H and 29AA9). Five out of the six samples produced sequences of high-enough quality for analysis, however, sample 29AA9 failed to produce a high-quality sequence read. For each of the successful samples a 983 bp and 565 bp region of the ITS and *cox*1 sequence amplicons were screened against reference sequences on the NCBI data base using the nucleotide basic local alignment tool (BLAST) and the results are shown in Table 4.

**Supplementary Fig. 1:**
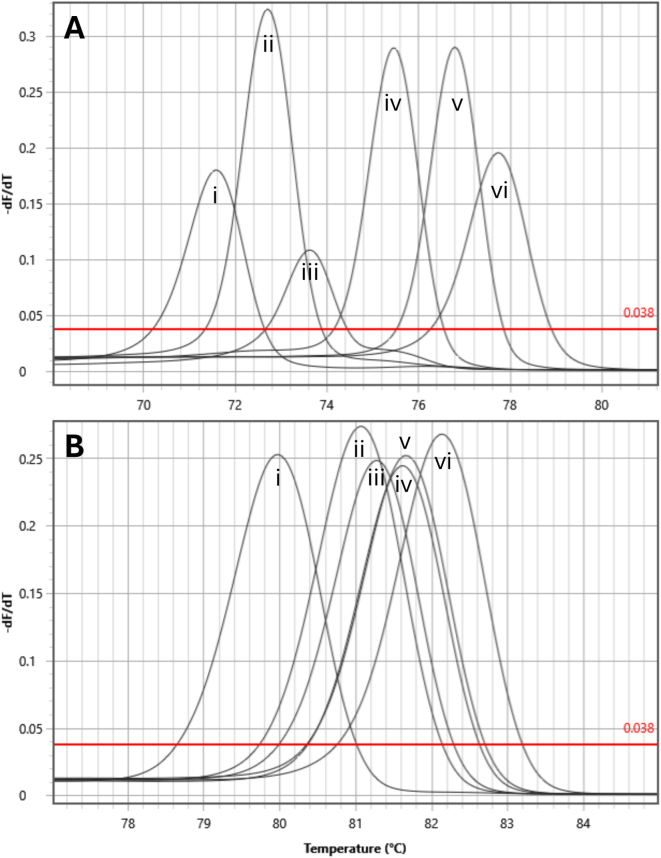
HRM profiles and species identification

Across all ITS sequences there were no heterozygous peaks observed and all five aligned closely with the *S. haematobium* reference sequences with a query and identity match of 100% and 99.9% respectively. The analysis of the *cox*1 sequences identified two *S. mattheei* sequences with a respective query cover and identity matches of 100% and 99.47% for sample Chimbende 31b and 100% and 99.82% for sample GPS-H. These results confirm these samples as first-generation hybrids between a female *S. haematobium* and a male *S. mattheei* worms. The remaining three *cox*1 sequences were identified as *S. haematobium,* with 100% query cover across all samples. Percentage identity varied, with samples Chimbende 33a and 33b having an identity score of 99.47% and no heterozygous peaks whilst sample Chimbende 31a possessed heterozygous peaks for *S. mattheei* and *S. haematobium* and had a lower identity score of 98.58%. Whilst samples Chimbende 33a and 33b are pure *S. haematobium* samples, sample 31a shows evidence of an ancestral hybridisation, Table 4 (Supplementary Fig. 1 and Fig. 2).

#### 3.3.4 Sanger sequencing of dissected adult worms

Of the 53 worms screened with the duplex mtDNA/nDNA real-time PCR six were selected for Sanger sequencing, three males and three females. As with the cattle FTA samples a 983 bp and 565 bp region of the ITS and *cox*1 sequence amplicons were screened against reference sequences on the NCBI data base using the basic local alignment tool (BLAST). The results identified all worms as *S. mattheei* with 100% query cover for both ITS and *cox*1 amplicons and an identity value of 99.9% and 99.8% respectively for all sequences submitted.

#### 3.3.5 Pilot GPS study and infection dynamics after treatment

**Fig. 3:**
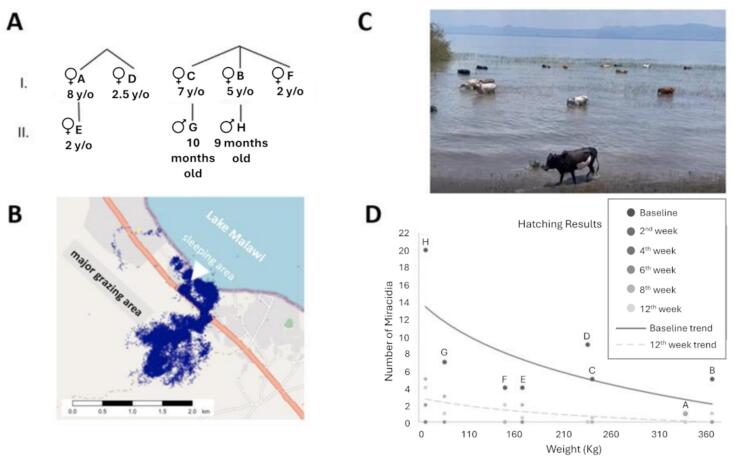
**A)** Pedigree of the cattle herd (HUGS A-H) that had GPS dataloggers fitted. Generations are numbered from the top of the pedigree in uppercase Roman numerals. Individuals in each generation are marked in letters (A-H). **B)** Map of the study area showing cumulative cattle movement (59,717 blue dots) over the three month period. The sleeping area of the cattle, a small wire fence coral is highlighted with white arrow with major grazing area depicted. Although grazing area changed through time, cattle movements are restricted to a 2-3 km area each day being constantly under supervision by their herder. Water contact times were estimated as cumulative GPS time in a buffer zone on the shoreline boundary (purple line) and lake itself (blue area). **C)** Photograph of cattle with the pilot GPS herd, mixed with other local animals, seen browsing on emergent vegetation. Of note infected intermediate snail hosts of schistosomiasis have been found at this site. Moreover, GPS cattle are in regular water contact with this site which is also a daily human water contact site for washing and swimming. **D)** Infection status, miracidia counts and intensity reduction rates over 12 weeks in eight cows naturally infected with schistosomes after single treatment with 40 mg/kg praziquantel. GPS data analysis was performed at LSTM and University of Washington. (For interpretation of the references to colour in this figure legend, the reader is referred to the web version of this article.)

At baseline, all of the eight GPS tracked animals were naturally infected with schistosomes, with the youngest animal GPS-H, noted to have a *S. haematobium-mattheei* hybrid. The egg count and the intensity of the infection showed that the youngest animal had the heaviest baseline infection before treatment. This pattern was mirrored in the reinfection rates with more miracidia hatched from the faeces of younger animals than older ones. Across a four-week, post-treatment, sampling period no hatched miracidia were observed in sampled cattle faeces. It was only in the sixth week post-treatment that the first positive cow was detected, which was cow H, the youngest cow in the herd. By the eighth week post-treatment, six out of the eight animals were positive by the faecal-egg hatching method (Fig. 3D).

A water-contact point on the shoreline was identified with the help of GPS tracking with the daily proportion of GPS points in the wet lake zone varying over time. In total, 59,717 GPS recorded locations were noted with an average of just under 20,000 recorded locations per month. After plotting these points, approximately 4% of GPS points overlap with the wet lake zone (Fig. 3). Analysis of GPS data revealed that the range of the cattle herd was surprisingly limited to the same five by five km^2^ area across the whole study.

**Fig. 4:**
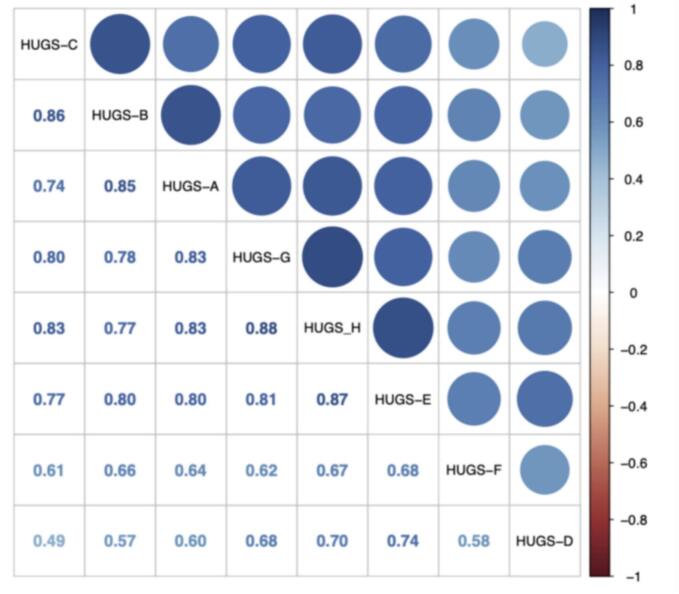
A correlation matrix between the eight GPS (HUGS A-H) tracked cattle daily movements highlight that some animals maintained closer spatial proximity to each other than other animals within the herd, a relationship likely based upon their immediate familiar pedigree. This shows the correlations between different animal's daily proportion of water contact GPS points via the size (larger is stronger correlation) and colour (blues correspond to a positive correlation) of the dot in the upper right half of the matrix. The lower left half of the matrix shows the corresponding correlation coefficient.”

The correlation matrix plot revealed that two of the animals' (D and F) water contacts were less correlated with other animals in the herd, Fig. 4. Cows D and F were recorded spending much more time away from the herd, but also away from each other. Analysis of results indicate that there were significant differences between animals' water exposure, however, this was not correlated to age and sex. Across the study period it was found that months were significantly associated with the herd's water-contact behaviour with the highest exposure occurring in April, followed by May, June then July.

## 4 Discussion

The emergence of *S. haematobium-mattheei* hybrids in human transmission in Malawi until now had an unknown aetiology but was suspected to arise from livestock, most likely in cattle, although natural primate hosts are also known [21]. Our investigation was the first formal survey to use faecal and carcass inspection to determine the prevalence of bovine schistosomiasis in Malawi. To the best of our knowledge, we also provide here the first evidence of *S. mattheei* infection in cattle in Malawi. The confirmed presence of *S. mattheei* infecting cattle in Malawi is a cause for concern since increased transmission could lead to significant animal health and economic impact, as well as a potential risk for hybridisation with *S. haematobium* [10].

By inspecting carcasses at slaughter it was possible to accurately determine the number of adult worms per cow, however, a disadvantage of this method was that the origin of the animal was unknown, since the slaughterhouses do not formally record where animals have originated. By contrast, locations of the cattle analysed by MHT were more explicit and have revealed a clear difference between Mangochi and Nsanje Districts, Fig. 1. The use of our novel real-time PCR HRM assay allowed for high numbers of samples to be rapidly screened, and also geolocated, then quickly identified for downstream genotyping and confirmation using DNA Sanger sequencing. The prevalence of hybrid infections was found to be 1.8% (3/166) with these infected animals being found in the Mangochi District at sites 4 and 7. The occurrence of a *S. haematobium-mattheei* hybrid and a *S. haematobium* infection in local cattle has now been confirmed. The latter is particularly important being the first to do so in SSA.

From our pilot GPS tracking findings, faecal egg counts were known before the treatment of the eight cattle from Mangochi District and it was found that the older animals had lower egg counts in their faecal samples. Although a decline in faecal egg counts with age may be expected in growing cattle as a result of greater egg dilution due to increasing faecal volumes [22]. It has been demonstrated experimentally [23] that the time-related decline in *S. mattheei* faecal egg excretion is mainly due to an immune-mediated suppression of fluke fecundity. Similar changes have been reported in cattle infections involving other schistosome species such as *Schistosoma spindale* [24]. In Zambia, about half of the eggs from *S. mattheei* from calves hatched in water, compared with only 15% of eggs from adult cows. The decline in egg output with age means that animals below 2 years of age perhaps play a more important role in the environmental contamination with viable eggs [22].

Whilst PZQ is not available in veterinary pharmacies locally, our PZQ treatment was effective in cows but as per human PZQ treatment of schistosomiasis, the effects of PZQ in cattle are not long-lasting. Given the re-infection patterns, retreatment is necessary within three months, which could potentially result in four rounds of treatment per calendar year. It should also be noted that not only the frequency of treatment is higher in cattle but also the volume of PZQ increases as a typical cow needs 25 PZQ tablets compared to only two and a half tablets for an average child. This is an important observation as simply put, there is insufficient PZQ available to treat adults and school age children [25,26], let alone commercial supplies to treat livestock. No doubt the deficit of PZQ is another factor adding to the entrenchment of the stubborn and chronic nature of bovine schistosomiasis here in Malawi. Despite the observed rapid reinfection rate in cattle, it appears the transmission of hybrids at this location and during this period of surveillance was rare.

The evidence from our GPS sub-study clearly shows the patterns and movements of animals along the lake, Fig. 3, Fig. 4, indicative of a daily repetitive cycle of grazing and watering subject to local resources. Since cattle are led as group by a herder, there are similarities in their water contact each day and there are several reasons why animals enter the lake, foremost for watering. First, these animals are also guided to reach several grazing parts on the shoreline which are blocked off by houses maintaining private land, often forcing them to walk along the shoreline such that an association between water contact and grazing points exists. Secondly, GPS tracking cattle movements throughout the three months has revealed that during dryer months inland cattle grazing was exhausted forcing cattle to scavenge feed upon emergent aquatic vegetation within the lake. Prior to this GPS study, few public health practitioners could have imagined foraging cattle in such deep water, yet this was exactly what we have documented quantitatively and qualitatively, (Fig. 3). With this increasing water contact, cattle contaminate the aquatic shoreline, infecting freshwater snails which may in turn infect people that make direct use of lake water.

Our project's One Health approach hopes to kick-start a much-needed appraisal of schistosomiasis in livestock [26], which is currently ignored in Malawi. Better integration of bovine epidemiology into medical epidemiology has some challenges [11,27]. Foremost there are no veterinary supplies of PZQ in Malawi, these exist only for medical purposes and the veterinary sector's current knowledge about bovine schistosomiasis is absent such that many human and animal health professionals are unfamiliar with the connection between human and animal schistosomes. More broadly, state involvement is limited in animal affairs, all the way from rearing to slaughter and the quality control of sold meats [28]. New strategies to control schistosome infection in livestock need to be developed, and quickly, if broader ambitions of disease control in humans are to be realised. An alternative or augmentation to treatment with praziquantel is to revive the old possibility of using vaccines, first explored using irradiated cercariae [29,30], and in this setting might have use in reducing hybrid transmission to manageable levels.

### 4.1 Study limitations

Our inspection of cattle at slaughter was hampered by poor institutional record keeping of these animals' rearing and origin. We are confident, nevertheless, to presume that the vast majority of cattle at slaughter in Blantyre originated from Chikwawa and/or Nsanje Districts and not from Mangochi District. Whilst genotyping miracidia obtained from MHT can better assign cattle to specific sampling locations the absence of extensive carcass sampling in Mangochi District is a limitation. Similarly, the detection of *S. haematobium* in certain animals, and only those at Chimbende, triggers a clear need for a later detailed inspection these individual cattle, for example, whilst still alive, with more intensive faecal and urine sampling to fully understand their environmental contaminating potential, or at slaughter, to ascertain precisely the anatomical location of these worms within the intestinal and/or urinary venous systems [31]*.* Whilst our inspection was confined to cattle, inspection of additional farmed or wild ungulate species within the area may well yield other potential zoonotic reservoirs.

## 5 Conclusion

We have documented and revealed an extensive burden of bovine schistosomiasis in southern Malawi and in so doing, confirmed that the *S. haematobium-mattheei* hybrid, although rare, is found in cattle, as is pure *S. haematobium* infection. The presence of pure *S. haematobium* in cattle indicates that these animals could potentially act as reservoir of urogenital schistosomiasis, raising concerns about of other potential animal reservoirs of this disease. We have also shown that treatment of cattle with PZQ is effective, but its impact is short-term. To safeguard zoonotic spillover from cattle to human transmission, we recommend additional methods of control need to be developed and explored.

The following are the supplementary data related to this article.

Supplementary material 1 Supplementary material 1

Supplementary material 2 Supplementary material 2

Supplementary material 3 Supplementary material 3

## Role of funding sources

The Hybridisations in UroGenital Schistosomiasis (HUGS) project receives funding from the 10.13039/100010269Wellcome Trust (220818/Z/20/Z).

## CRediT authorship contribution statement

**Alexandra Juhász:** Data curation, Formal analysis, Investigation, Writing – original draft, Writing – review & editing. **Peter Makaula:** Formal analysis, Investigation, Methodology, Supervision, Writing – original draft, Writing – review & editing. **Lucas J. Cunningham:** Data curation, Formal analysis, Investigation, Methodology, Writing – original draft, Writing – review & editing. **Sam Jones:** Data curation, Formal analysis, Investigation, Methodology, Writing – review & editing. **John Archer:** Investigation, Methodology, Writing – review & editing. **David Lally:** Data curation, Investigation, Methodology, Writing – review & editing. **Gladys Namacha:** Investigation, Methodology, Writing – review & editing. **Donales Kapira:** Investigation, Methodology, Writing – review & editing. **Priscilla Chammudzi:** Investigation, Methodology, Writing – review & editing. **E. James LaCourse:** Data curation, Formal analysis, Methodology, Writing – review & editing. **Edmund Seto:** Investigation, Software, Visualization, Writing – review & editing. **Sekeleghe A. Kayuni:** Data curation, Investigation, Methodology, Project administration, Writing – review & editing. **Janelisa Musaya:** Conceptualization, Data curation, Funding acquisition, Methodology, Supervision, Validation, Writing – original draft, Writing – review & editing. **J. Russell Stothard:** Conceptualization, Formal analysis, Funding acquisition, Investigation, Methodology, Project administration, Supervision, Validation, Writing – original draft, Writing – review & editing.

## Declaration of competing interest

The authors report no conflicts of interest.

## Data Availability

Data will be made available on request.
