# Supplementary material for: Revealing bovine schistosomiasis in Malawi: Connecting human and hybrid schistosomes within cattle
Source: One Health. 2024 Jun 14;19:100761. doi: 10.1016/j.onehlt.2024.100761 (PMC11253675; doi:10.1016/j.onehlt.2024.100761)
Supplement: Supplementary material 1 [file mmc2.docx]

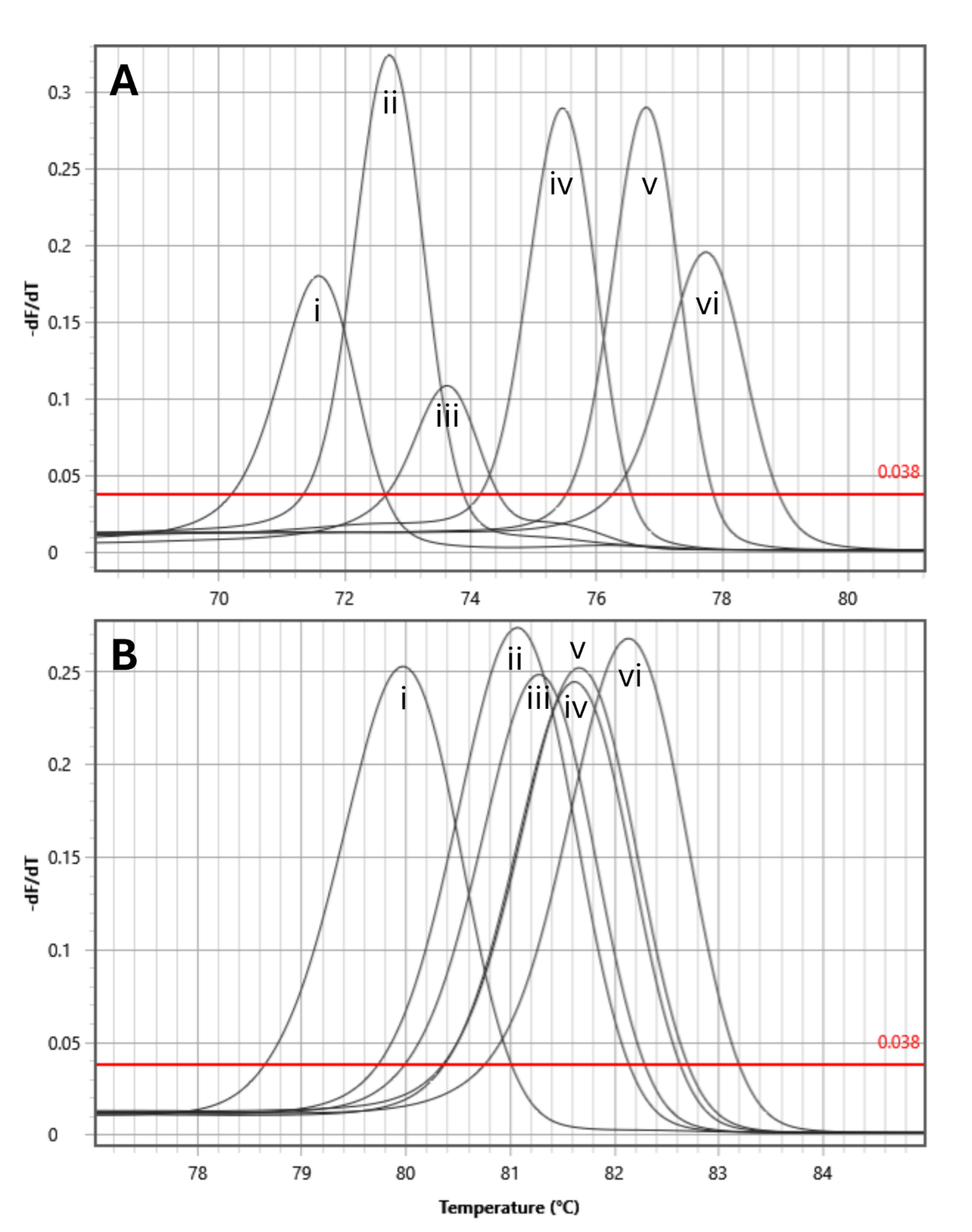


**Supplementary Fig 1.** Melt peak profiles for mtDNA qPCR (A) for the following species: *S. mattheei* (i), *S. curassoni* (ii), *S. bovis* (iii), *S. haematobium* (iv), *S. mansoni* (v) and *S. margrebowiei* (vi). The melt profile for the nDNA qPCR (B) is also depicted for the following species: *S. mansoni* (i), *S. mattheei* (ii), *S. margrebowiei* (iii), *S. curassoni* (iv), *S. bovis* (v) and *S. haematobium* (vi).
