# Supplementary material for: Revealing bovine schistosomiasis in Malawi: Connecting human and hybrid schistosomes within cattle
Source: One Health. 2024 Jun 14;19:100761. doi: 10.1016/j.onehlt.2024.100761 (PMC11253675; doi:10.1016/j.onehlt.2024.100761)
Supplement: Supplementary material 3 [file mmc4.pptx]

## Slide 1
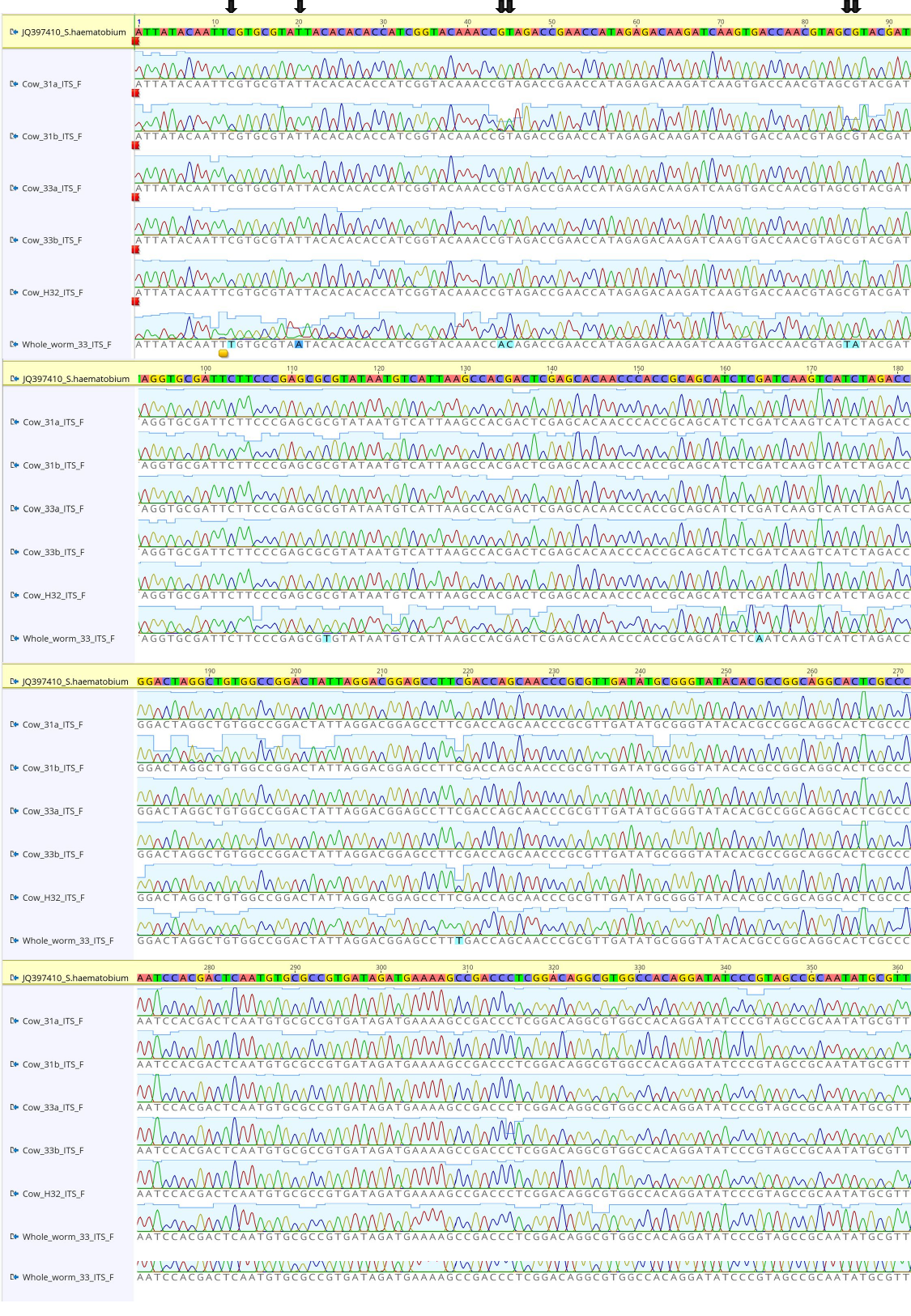

## Slide 2
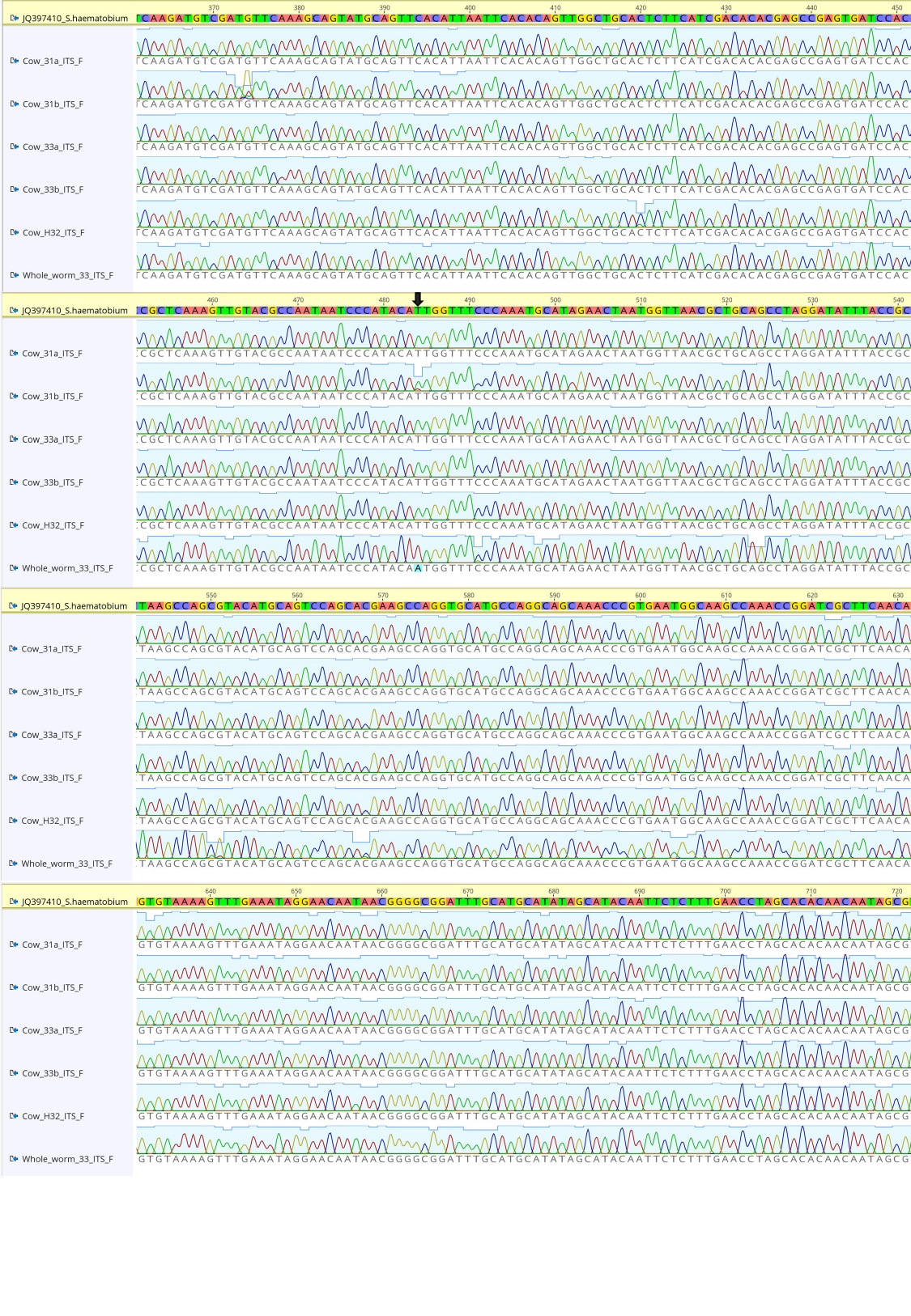

## Slide 3
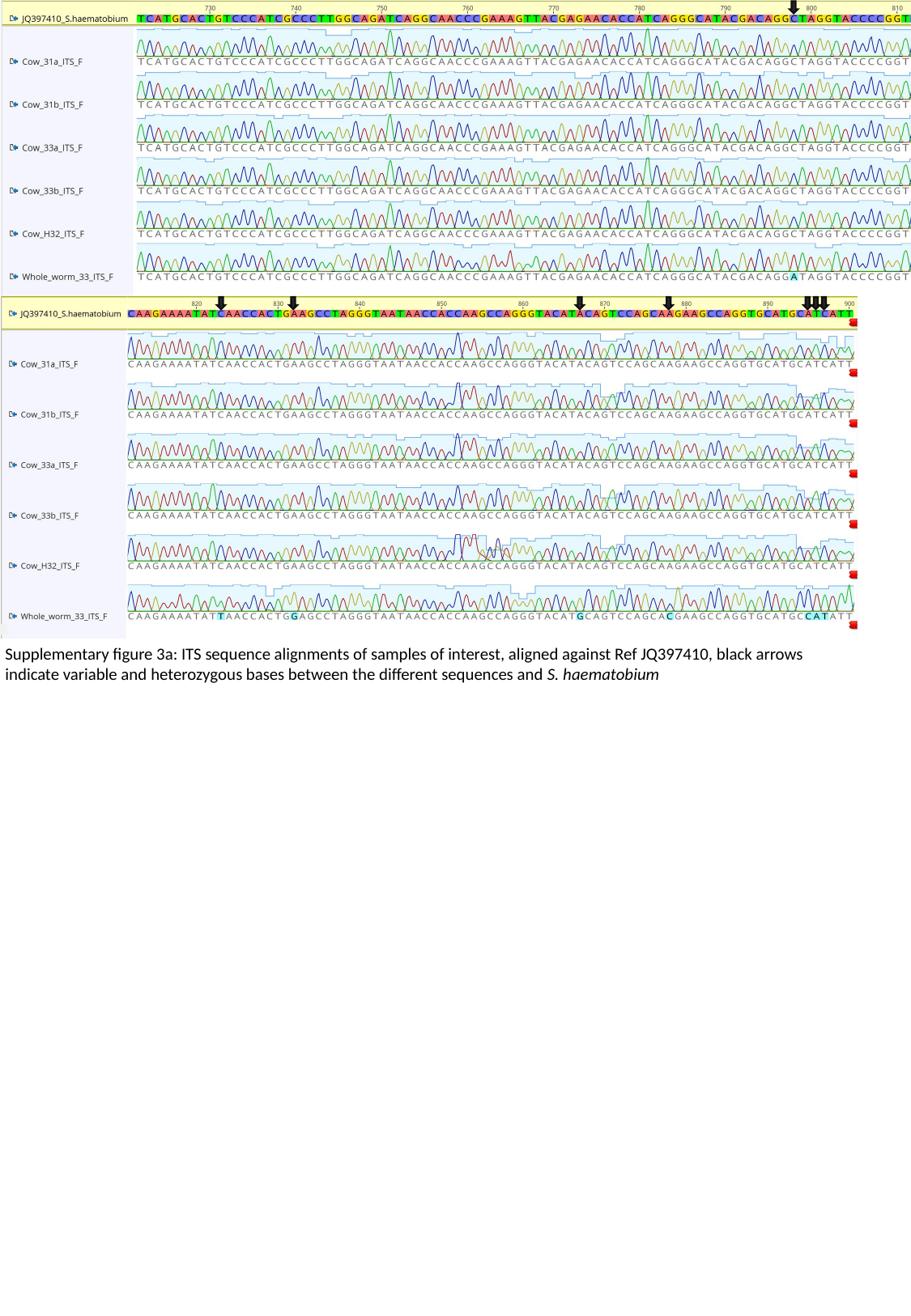

Supplementary figure 3a: ITS sequence alignments of samples of interest, aligned against Ref JQ397410, black arrows indicate variable and heterozygous bases between the different sequences and S. haematobium

## Slide 4
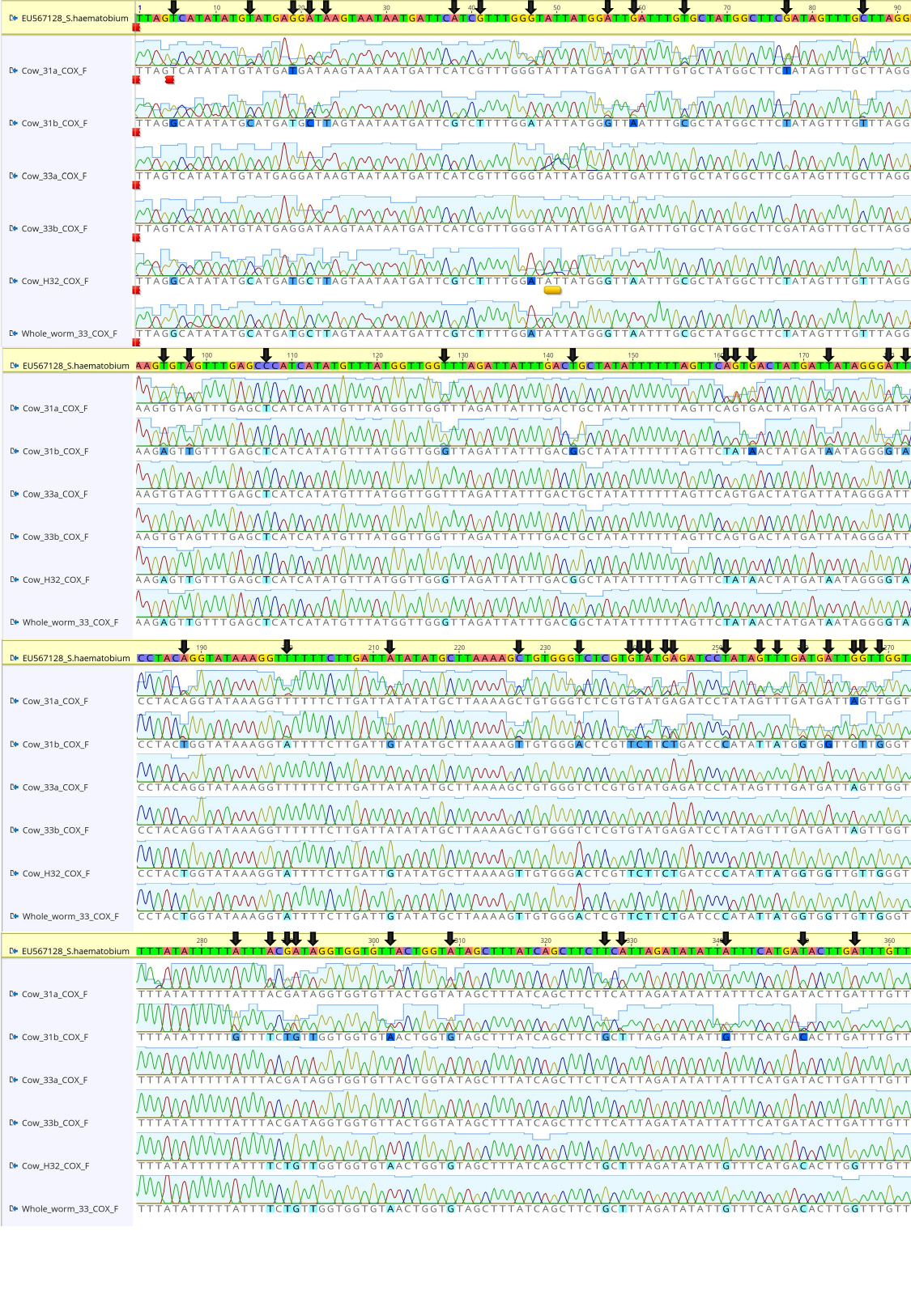

## Slide 5
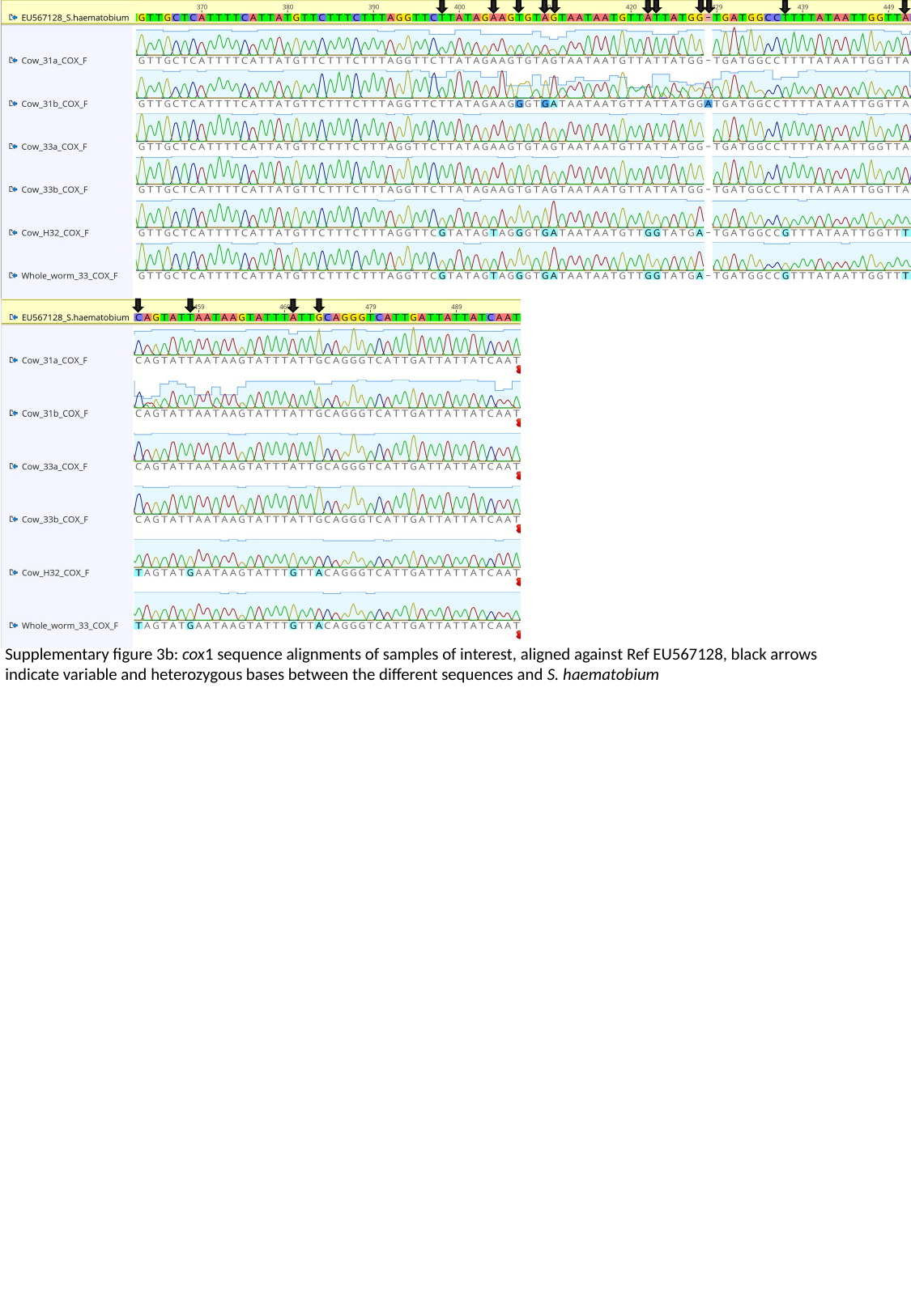

Supplementary figure 3b: cox1 sequence alignments of samples of interest, aligned against Ref EU567128, black arrows indicate variable and heterozygous bases between the different sequences and S. haematobium
